# Why Human Papillomaviruses Activate the DNA Damage Response (DDR) and How Cellular and Viral Replication Persists in the Presence of DDR Signaling

**DOI:** 10.3390/v9100268

**Published:** 2017-09-21

**Authors:** Molly L. Bristol, Dipon Das, Iain M. Morgan

**Affiliations:** 1 VCU Philips Institute for Oral Health Research, Virginia Commonwealth University School of Dentistry, Department of Oral and Craniofacial Molecular Biology, Richmond, VA 23298, USA; mlbristol@vcu.edu (M.L.B.); ddas@vcu.edu (D.D.); 2 Massey Cancer Center, Virginia Commonwealth University, Richmond, VA 23298, USA

**Keywords:** HPV, human papillomavirus, replication, initiation, life cycle, DNA damage response, *TopBP1*, E1, E2, ATM (ataxia-telangiectasia mutated), ATR (ataxia telangiectasia and Rad3 related), DNA damage signaling, cervical cancer, head and neck cancer, homologous recombination, MRN (Mre11-Rad50-Nbs1)

## Abstract

Human papillomaviruses (HPV) require the activation of the DNA damage response (DDR) in order to undergo a successful life cycle. This activation presents a challenge for the virus and the infected cell: how does viral and host replication proceed in the presence of a DDR that ordinarily arrests replication; and how do HPV16 infected cells retain the ability to proliferate in the presence of a DDR that ordinarily arrests the cell cycle? This raises a further question: why do HPV activate the DDR? The answers to these questions are only partially understood; a full understanding could identify novel therapeutic strategies to target HPV cancers. Here, we propose that the rapid replication of an 8 kb double stranded circular genome during infection creates aberrant DNA structures that attract and activate DDR proteins. Therefore, HPV replication in the presence of an active DDR is a necessity for a successful viral life cycle in order to resolve these DNA structures on viral genomes; without an active DDR, successful replication of the viral genome would not proceed. We discuss the essential role of *TopBP1* in this process and also how viral and cellular replication proceeds in HPV infected cells in the presence of DDR signals.

## 1. Introduction

Human papillomaviruses (HPV) have double stranded DNA genomes of around 8 kb and productively infect only the epithelium. There are hundreds of HPV types and they are broken into two categories: high risk HPV (HR-HPV) and low risk HPV (LR-HPV), based on their roles in human disease [1]. LR-HPV cause benign warts but can be problematic; for example, genital warts are debilitating and have an associated morbidity for the afflicted individual, with HPV6 and HPV11 being responsible for around 90% of genital warts. HR-HPV are so called due to their ability to cause cancer. While most HR-HPV infections are resolved by the host immune system and do not result in any disease, a small fraction of infected individuals have persistent infections that can progress to cancer. All cancers of the ano-genital regions have a high incidence of HR-HPV presence including cervical (almost all have HR-HPV), anal, penile, and vulvar cancers. The other major anatomical site where HR-HPV cause cancer is in the oropharyngeal region (HPV + OPC, oropharyngeal carcinoma), although HR-HPV positive tumors can be found throughout the oral cavity [2,3,4]. The incidence of HPV + OPC has risen substantially in the past two decades and represents a current and ongoing health emergency as there are currently no diagnostics (such as Pap smears that assist with the management of cervical cancer) that can be used to manage this disease. While the current prophylactic vaccines targeting both LR-HPV and HR-HPV will reduce the disease burden on future generations, it remains a priority to develop therapeutics targeting these viruses for several reasons. Firstly, the vaccines are only prophylactic, and therefore, those already infected (the large majority of the population) remain at risk of disease. Secondly, in developed nations, many have failed to successfully introduce this vaccine; in 2014 in the United States, only 44% of girls and 25% of boys received the recommended vaccine doses. Thirdly, it is possible that over the decades the viruses targeted by the vaccines may evolve to evade vaccine targeting and therefore persist in causing disease. Fourthly, in the developing world where most of the HR-HPV deaths occur, there has been a limited ability to introduce the prophylactic vaccine for financial and/or logistical reasons. Novel therapeutic approaches to combat HPV infections are therefore required and enhancing our understanding of the HPV life cycle will assist in identifying such approaches. One such target is viral replication.

## 2. HPV DNA Replication

HPV infect the basal layers of the stratified epithelium following tissue abrasion, and during cellular division, the viral life cycle starts [5]. Once located in the nucleus, the viral genome is bound by cellular factors to activate transcription from the viral genome via the long control region (LCR), the only non-coding segment of the viral genome [6]. This results in the production of a transcript that is then spliced to produce the viral proteins required to instigate a successful infection. The oncogenes *E6* and *E7* are expressed and target p53 and pRb respectively for degradation, resulting in the promotion of cellular proliferation. The viral replication factors E1 and E2 are also expressed. E2 acts as an origin recognition factor; there are four 12bp palindromic E2 target sequences within the LCR and E2 can bind all of these [7]. Three of the target sites surround the A-T rich origin of replication and once located there E2 can recruit the E1 helicase to the origin of replication via a protein-protein interaction [8]. The viral proteins interact with a host of factors that all contribute to viral replication initiation and completion (see below). This initial phase of the viral life cycle is called establishment and the viral genome copy number accumulates to 20–50 copies per cell. As the infected cell proliferates, it begins to move up through the epithelium and starts to undergo the differentiation process; the viral genome copy number remains at 20–50 per cell and this stage of the viral life cycle is termed maintenance. In the upper layers of the epithelium, following differentiation of the host cell and the cessation of host cell DNA replication, the virus undergoes an amplification stage where the viral genome copy number increases to around 1000 copies per cell. At this time, the L1 and L2 proteins are also produced and the viral genome is then encapsulated into viral particles that egress from the upper layers of the differentiated infected tissue. Therefore, there are three stages to the viral life cycle with regards to DNA replication; establishment, maintenance, and amplification. The precise cellular proteins and mechanisms used to control these three different phases remain unclear.

Therapeutic targeting of LR-HPV DNA replication has been achieved using small molecule inhibitors of the E1–E2 interaction [9,10,11]. The inhibitors developed did not work well with HR-HPV, presumably due to the differences in the E1–E2 interactions between the different viruses. The discovery that, in cervical cancer, the viral genome is often integrated into the host genome (therefore losing any requirement for E1–E2 function) resulted in a loss of enthusiasm for targeting HR-HPV DNA replication as a therapeutic strategy. [12,13,14,15,16]. However, we believe this is an oversight for several reasons. The primary one is that in HPV + OPC, around 90% of which are caused by HPV16, the virus remains episomal in a large number of cases and is likely replicating in an E1–E2 dependent manner. Two-thirds to three quarters of these tumors express the E1 and E2 proteins and at least 40% have purely episomal viral genomes [17,18,19]. Our recent investigations suggest that in fact up to three quarters of HPV + OPC are replicating episomal genomes (some of them viral-human hybrids) in an E1–E2 dependent manner with no integrated virus [20]. Also, in cervical cancers (around 50% of which are caused by HPV16), there are a significant minority that retain episomal viral genomes replicating in an E1–E2 dependent manner. Targeting this replication in these tumors would reduce the viral genome load and therefore the expression levels of the viral oncogenes *E6* and *E7*, and such a reduction is known to reactivate the p53 and pRb pathways inducing senescence and/or apoptosis [21,22,23,24,25,26,27,28,29,30]. Therefore, for a significant number of HR-HPV cancers, targeting E1–E2 DNA replication remains a viable therapeutic strategy. In order to be able to effectively target this process, we must understand it better.

## 3. HPV Replication Activates the DDR

Several labs have demonstrated the activation of the DDR by HR-HPV (from now on mostly HR will be discussed and therefore only HPV will be used unless stated otherwise) in HPV containing epithelium [31,32,33,34,35,36,37,38,39,40,41,42,43]. This activation is not required to maintain the viral genomes in epithelial cells, but is necessary for the amplification stage of the viral life cycle. It is not known whether the establishment phase of the viral life cycle requires the activation of the DDR. Both ATM (ataxia-telangiectasia mutated) [34] and ATR (ataxia telangiectasia and Rad3 related) [33,44,45] are activated by HPV. The details of the ATM and ATR pathways will not be described here as there are many excellent reviews available. Suffice to say that the activation of these kinases by DNA damaging agents results in signaling cascades that ordinarily results in cell cycle arrest to promote the repair of any DNA damage. In a “healthy” cell, cell cycle re-start will only occur following the repair of the damaged DNA. However, in HPV containing cells, the cell cycle progresses with the DDR turned on and viral DNA replication continues. Therefore, the virus has evolved mechanisms to turn on the DDR but allow cell cycle progression along with both host and viral DNA replication. These observations raise a host of questions. How and why do HPV activate the DDR? Is the HPV induced DDR distinct from that induced by other damaging agents? How does viral and host replication proceed in the presence of the HPV induced DDR? The answers to these questions are important as the HPV induced DDR may represent a therapeutic target to selectively disrupt this process in HPV transformed cells; such disruption could block viral and/or host replication, resulting in cell death. There have been several recent and excellent reviews on the DDR and the HPV life cycle and it is not our intention to cover the same ground again [46,47]. Rather, we will focus on how the viral replication factors contribute to the activation of the DDR, why they do this, and how viral and cellular replication proceeds in the presence of an activated DDR.

## 4. The Role of Viral Replication Proteins in Activating the DDR

**Figure 1:**
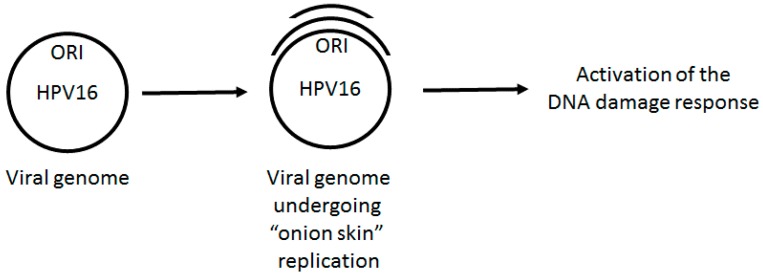
HPV E1–E2 mediated replication has the capacity to directly activate DNA damage repair pathways. Following infection, the viral genome must be quickly amplified to 20–50 copies per cell. This replication potentially generates “onion skin” structures causing torsional stress promoting the formation of single stranded DNA and double strand breaks that would be recognized as DNA damage by the cell. Therefore, during the establishment phase of the cell cycle, we propose that HPV replication would activate a local DDR via aberrant DNA structures on the viral genome. Failure to activate this response would result in failure to replicate.

The E1 protein can interact with a host of cellular proteins involved in DNA replication, including components of DNA polymerase α, Topoisomerase 1, and the single stranded binding protein replication protein A [48,49,50,51,52,53]. It is of course logical that a viral replication protein should interact with such factors. However, the over expression of E1 can also activate the DDR and the mechanism for this activation is reasonably clear [41,43,45]. The first clue that E1 (and E2) is involved in the DDR in mammalian cells came following the analysis of E1/E2 replication from integrated HPV16 and HPV18 genomes in cervical cancer cell lines [31]. The over expression of the E1 and E2 proteins (the expression of which is lost in the cervical cancer lines used in this study due to an integration event) initiated replication from the viral origins of replication within the integrated viral genomes. Because E1–E2 replication is not limited to a once and once only per cell cycle rule (it cannot be as it has to establish viral genome counts of 20–50 per cell following infection and also amplify to 1000 copies per cell in the differentiated epithelium), the replication from the integrated origin fires repeatedly. This results in an “onion skin” type of replication [42,54,55,56,57,58] that presents structural DNA problems that must be managed in order to execute a successful viral life cycle (Figure 1). These aberrant structures result in the formation of DNA damage repair foci in the E1/E2 over expressing cells that consist of a host of DDR and repair proteins along with replication factors. The factors involved in this process include the MRN (Mre11-Rad50-Nbs1) complex, ATM, *Chk2*, and also the enhanced phosphorylation of *Chk2*, indicating the activation of ATM along with the generation of γH2AX [42]. These studies were based around “forcing” E1–E2 replication from integrated origins of replication in cervical cancer cell lines. Strikingly, the formation of these foci was also observed in differentiating HPV31 cells alongside the activation of the DDR [34]. In this study, the inhibition of the DDR via ATM inhibitors did not abrogate the maintenance of viral DNA replication, but did block the amplification of the viral genome in the differentiating epithelium. The question of how the replication foci in the HPV31 containing cell lines form has not been clearly answered, but the results from the experiments over expressing E1–E2 in cell lines with integrated viral genomes presents a clue. It seems entirely possible that, during the establishment phase of the viral life cycle, the initial burst of replication from the viral origins results in the equivalent of “onion skin” like structures on the viral episomal genomes being replicated. Such structures would activate the DDR and recruit the DDR factors required to resolve such structures. For the sake of the viral life cycle, this would be essential as “onion skin” replication intermediates would be detrimental to the efficient establishment of episomal viral genomes in the infected cells. It should also be noted that replication intermediates generated by “onion skin” replication would potentially generate both double stranded DNA breaks along with stretches of single stranded DNA. The former is a classic activator of the ATM pathway, while the latter is a signal of replication stress that activates the ATR signaling pathway. Both of these pathways are activated in HPV containing keratinocytes and both contribute towards the viral life cycle. Therefore, during the initial phase of viral infection, the actual process of viral DNA replication has the capacity to activate the DDR due to the formation of aberrant DNA structures on the replicating viral genomes. Over expression of the E1 protein by itself, as well as when complexed with E2 and an origin of replication, can induce a DDR. This is likely due to the indiscriminate interaction of E1 with the host chromatin, resulting in inappropriate genome unwinding during the S phase and in torsional stress and DNA structures that would then activate the ATM and ATR DDR pathways.

The E7 protein has also been implicated in the activation of the DDR in HPV positive cells [34,40]. E7 can increase the levels of a number of host DDR proteins in two ways. Firstly, via disturbance of the E2F1-pRb interaction allowing E2F1 to activate transcription of *DDR* genes, and secondly, via a direct interaction between E7 and DDR factors that stabilizes the host proteins using an as yet unknown mechanism. Due to this enhanced elevation, E7 also increases the levels of activated DDR factors such as phosphorylated *Chk2* and *Chk1*. However, even though E7 can directly complex with several DDR factors, it is not clear whether it actually activates the DDR. Clearly, E7 amplifies the DDR via increasing the expression of DDR proteins, but to date, there have been no quantitative studies investigating whether E7 can actually directly activate the enzyme activity of DDR kinases. Similarly, the E6 protein can clearly regulate the cellular response to the DDR via manipulation of p53 protein levels, for example, but a direct role for this protein in activating the DDR is not clear. A more likely explanation for the enhanced DDR in HPV positive cells is that E1 activates the DDR, while E7 increases the levels of proteins involved in the DDR.

## 5. Formation of the E1–E2 Replication Complex Promotes the Activation of the ATM and ATR DDR Pathways: The Role of *TopBP1*

From the section above, we propose that the activation of the DDR in HPV positive cells originates from the replication of the viral genome by E1 and E2 in a more than once per cell cycle manner, resulting in aberrant DNA structures recognized by the cell as DNA damage, therefore activating the DDR. This signal is also amplified by the E7 protein, increasing the levels of components of the DDR. Here, we discuss in more detail the possible mechanisms that result in the activation of the DDR by HPV E1–E2 replication.

**Figure 2:**
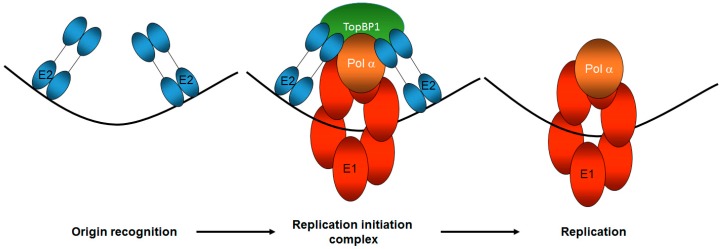
*TopBP1* is a key component of correct HPV E1–E2 mediated DNA replication. The E2 protein recognizes the viral origin of replication (represented by the black line) and therefore serves as the viral origin recognition complex. It then recruits a host of proteins to the origin via protein-protein interactions, including the viral helicase E1 and *TopBP1*, to form the replication initiation complex. *TopBP1* can interact with both E2 and E1 and is known to recruit DNA polymerases to mammalian replication complexes. Following the initiation of replication, the E1 helicase and host polymerases can then begin to replicate the viral genome. The number and function of host proteins required for this process remains unclear. Only one copy of the di-hexameric E1 complex required for replication is shown for the sake of simplicity.

Several years ago, we demonstrated that the HPV16 E2 protein interacts with the cellular DNA replication, damage, and repair factor *TopBP1* [59]. In yeast and Xenopus model systems, *TopBP1* (and homologues) is required for interacting with origin recognition complex proteins and loading *Cdc45* and the *GINS* complex (*Sld5*, *Psf1*, *Psf2*, *Psf3*) onto *MCM2-7* in an S phase kinase specific manner at the *G1-S* transition to form the replication helicase, *CGM* [60,61,62,63,64,65,66]. Therefore, *TopBP1* is an attractive candidate for mediating E1–E2 initiation of replication and this is supported by the observation that mutants of E2 that fail to interact with *TopBP1* in an optimum fashion are compromised in replication, and when introduced into the viral genome, abrogated the viral life cycle in primary keratinocytes [67,68]. Therefore, the interaction between E2 and *TopBP1* is essential for the viral life cycle due to an essential role in the initiation of DNA replication (Figure 2). We also recently demonstrated that E1 interacts with *TopBP1* independently of E2 [41]. *TopBP1* has nine BRCT (*BRCA1* carboxyl terminal) domains that serve as hydrophobic pockets that can interact with damaged DNA structures, phosphor-proteins, and proteins [69]. No cellular protein has so many domains and *TopBP1* can act as a scaffold, allowing interactions with a host of cellular factors simultaneously; it is involved in all aspects of nucleic acid metabolism including replication, transcription, DNA damage recognition, signaling, and repair [70].

**Figure 3:**
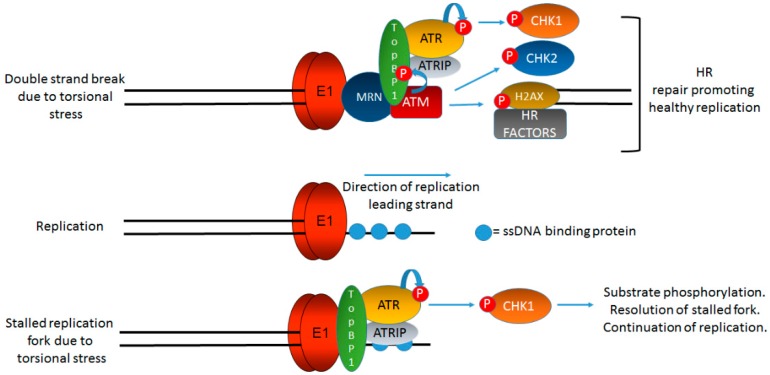
*TopBP1* is a key protein initiating and coordinating DDR signaling at HPV replication centers. Figure 1 indicates that onion skin DNA replication can be formed on HPV genomes during the initial phases of infection and we propose that this would activate the DDR. Figure 2 briefly summarizes the role of *TopBP1* in coordinating the initiation of HPV replication. In this figure, we demonstrate the key role of *TopBP1* in both initiating and controlling DDR signaling at HPV replication forks. Torsional stress during onion skin replication would generate both stretches of single stranded DNA (bottom panel) and also double strand breaks (top panel). Both of these DNA structures would generate proposed localized DDR signaling, and *TopBP1* is key to the activation of both ATR and ATM. HPV replication in the absence of *TopBP1* is highly mutagenic, demonstrating that it is a key component of the HPV replication process. The precise order, sequence, function, and components of HPV replication remain to be fully elucidated. The black lines represent DNA strands. The blue “circular” line represents phosphorylation events. See text for details.

*TopBP1* can interact with sites of replicative stress and we propose that the E1–E2 replication can generate such DNA structures (Figure 1) and help retain *TopBP1* at the viral genome, although the direct interaction between E1 and *TopBP1* and E2 and *TopBP1* to facilitate viral DNA replication initiation could also play a role in recruitment. Chromatin immunoprecipitation experiments show that *TopBP1* is located at E1–E2 replication complexes, demonstrating the localization of *TopBP1* to the viral genome without external genotoxic stress [68]. As well as the E1–E2 interaction, there are several mechanisms that would assist in the recruitment and retention of *TopBP1* to sites of DNA damage via the recognition of aberrant DNA structures generated during E1–E2 replication (including single stranded DNA and transient double stranded breaks on the viral genome). MRN can recognize breaks in double stranded DNA and recruit both ATM [71] and ATR [72], as well as *TopBP1*. ATRIP can recognize single stranded DNA and assist with the recruitment of both ATR and *TopBP1* to sites of DNA damage [73]. The interaction between *TopBP1* and the ATRIP-ATR complex activates the catalytic activity of ATR, therefore activating the DDR [73,74,75,76,77,78]. *TopBP1* does not activate ATM but is a substrate for this protein, and the phosphorylation of *TopBP1* by ATM promotes the ability of *TopBP1* to stimulate the ATRIP-ATR complex, and therefore, ATM indirectly stimulates ATR activity via *TopBP1* phosphorylation [79]. The mechanism for ATM activation by HPV replication is likely related to double strand breaks forming at the replication origins due to replication stress. These double strand breaks recruit the MRN (Mre11-Rad50-Nbs1) complex which is required for the recruitment and activation of ATM and it is well known that the MRN complex is recruited to sites of HPV replication [34,35,39]. The mediator of DNA damage checkpoint 1, MDC1, which binds Mre11 at double strand breaks, recruits ATM to the sites of double strand breaks [80,81]. Therefore, in HPV replication, double strand breaks would recruit the MRN complex. This would then recruit ATM to the HPV replication centers, resulting in activation which then stimulates ATR activity via *TopBP1* phosphorylation. This pathway explains how HPV activate the DDR via replication complexes. It is also of note that *TopBP1* interacts with the MRN complex which promotes the phosphorylation of *TopBP1* by ATM; therefore, *TopBP1* likely plays a key role in stabilizing the HPV DNA replication foci [72,82]. Figure 3 summarizes how single stranded DNA and double strand breaks on the viral genome promote the activation of DDR signals that resolve these structures to promote healthy replication.

Supporting a key role for *TopBP1* in viral replication, the shRNA targeting of *TopBP1* destroys E1–E2 replication foci, demonstrating the core requirement for this protein in the formation of these foci [41]. This work was carried out in C33a cells, a cervical cancer cell line, and these cells survived for around three days before dying due to catastrophic chromatin fragmentation. The levels of E1–E2 replication in the absence of *TopBP1* remained unchanged from control cells; however, the replication that occurred was highly mutagenic. We have tried to repeat this in primary cells but *TopBP1* depletion in these cells results in G1 arrest, and therefore, it is not possible to do these experiments. Transgenic mice with the targeted knock out of *TopBP1* do not survive long into embryogenesis [83]. It is not surprising that *TopBP1* is an essential gene given the role that the protein has in replication and DNA repair as described above. *TopBP1* is also a transcription factor, and regulates the transcriptional activity of both E2F1 and p53 via a variety of mechanisms [84,85]. Our E2 mutant that has a compromised interaction with *TopBP1* and has compromised replication can activate and repress transcription. Therefore, we do not think that *TopBP1* is a critical regulator of E2 transcription function [67].

To date, we describe a mechanism that explains the activation of the DDR by E1–E2 mediated DNA replication. The purpose of this activation is to resolve damaged DNA structures generated during viral replication. We propose that the purpose of this DDR in the context of E1–E2 replication is to promote the homologous recombination of the replicating viral genomes and *TopBP1* would play an essential role in this process as it has been shown to be involved in HR [70]. It is also of note that in addition to the MRN complex, other HR proteins are recruited to the viral replication complex [35] and *TopBP1* acts as a scaffold to stabilize the HR proteins and enhance the efficient resolution of any DNA damage on the virus. Using this mechanism, the local activation of the DDR by HPV E1–E2 replication would promote high fidelity replication of the viral genome; it is of note that E1–E2 replication in the absence of *TopBP1* is highly mutagenic [41].

The role of *TopBP1* in regulating viral genome replication and the generation and response to DDR signaling demonstrates that this protein is essential for both the establishment and maintenance phases of the HPV life cycle. *TopBP1* can also act as a restriction factor for HPV replication during the maintenance phase of the cell cycle; knockdown of *TopBP1* elevates the viral genome copy number in model systems, while over expression represses replication [86]. Therefore, *TopBP1* is a key factor regulating HPV replication at all phases of the viral life cycle.

## 6. The Role of *SIRT1* in the Viral Life Cycle

While we have demonstrated an essential role for *TopBP1* in the viral life cycle, we wanted to expand this to investigate the role of *TopBP1* interacting partners in HPV replication that exhibited enzyme activity. The enzymatic activity of such factors could then potentially be targeted to disrupt the viral life cycle. One such factor is the deacetylase *SIRT1* [87]. Glucose starvation activates *SIRT1* which then deacetylates *TopBP1* resulting in disruption of the *TopBP1*-Treslin complex that is required for the initiation of DNA replication [88]. Conversely, DNA damage blocks *SIRT1* deacetylation of *TopBP1* and this results in a *TopBP1*-Rad9 interaction that promotes ATR activation [89]. Therefore, these signals (metabolic and DNA damage stress) have the opposite effects on the *SIRT1*-*TopBP1* interaction, but both ultimately result in cell cycle arrest. To investigate the role of *SIRT1* in regulating E1–E2 DNA replication, we generated CRISPR clones not expressing *SIRT1*. In these clones, E1–E2 replication was slightly enhanced and the acetylation of E2 was greatly increased, resulting in the stabilization of this viral replication protein [90]. This enhanced E2 level would explain the increased DNA replication. *SIRT1* is recruited to the HPV16 origin of replication in an E1–E2 dependent manner, and therefore, the results suggest that *SIRT1* is recruited to the replication complex in order to deacetylate E2 and keep replication levels under control. It is also of note that the *SIRT1* substrate *TopBP1* also acts as a restriction factor for HPV replication [86]. More recently, we have demonstrated that in the absence of *SIRT1*, E1–E2 replication is extremely mutagenic and the E1–E2 DNA replication foci are disrupted in the absence of *SIRT1* (Das, Bristol and Morgan, unpublished). While we were working on this project, the Laimins lab published a report on the role of *SIRT1* in the HPV31 life cycle [91]. They demonstrated that *SIRT1* is over expressed in HPV31 immortalized keratinocytes and that when *SIRT1* levels are reduced using shRNA targeting, the viral genome is depleted in the HPV31 immortalized keratinocytes. These results agree with our observations. In our assays, although depletion of *SIRT1* does not reduce E1–E2 replication, it clearly results in very poor quality replication. In the report from Laimins, long term culture with reduced *SIRT1* expression reduces the viral genome levels and our results suggest that this is likely due to the very poor quality replication of the viral genome, resulting in viral genome loss in the long term culture. Therefore, it seems likely that *SIRT1* is absolutely required for the establishment phase of the viral life cycle to ensure high fidelity replication. Whether its interaction with *TopBP1* is required to promote high fidelity HPV replication is currently under investigation. Another striking feature of *SIRT1* is that it can also regulate homologous recombination pathways. *SIRT1* is required for the formation of DNA damage response foci in mammalian cells and deacetylates Nbs1, providing a substrate for ATM which then phosphorylates Nbs1 on S278 and S343 [92]. The MRN complex is then recruited to sites of damage and the HPV E1–E2 replication foci [35]. Therefore, the absence of *SIRT1* promoting mutagenic replication in the absence of large replication foci could be due to persistent Nbs1 acetylation preventing ATM phosphorylation and the recruitment of the MRN complex. In addition, there is also evidence that *SIRT1* contributes to the regulation of homologous recombination independently of the MRN complex [93]. We propose that *SIRT1* is a key enzyme in the regulation of the viral life cycle via its role in the regulation of HR and therefore viral DNA replication.

## 7. Why Do the DDR Signals Generated by HPV Not Arrest Viral Replication or Host Replication?

Activation of the DDR with progress of replication is not the normal response to signaling from this pathway. Ordinarily, DNA damaging agents activate the ATM and ATR pathways in order to send several signals to the cell: DNA replication is arrested, cell cycle progression is arrested, DNA damage repair pathways are activated, DNA repair occurs, and DNA replication and cell cycle progression are then re-started [94]. Under ideal circumstances, this mechanism allows the damaged cell to retain a healthy genome with no accumulation of potentially cancer causing mutations. So how does viral and cellular replication proceed in the presence of the HPV generated DDR? For cellular replication, the answer is likely a combination of mechanisms. The first possible mechanism relates to the localization of the DDR generated by HPV. The foci generated by HPV, either in transient E1–E2 replication assays or in HPV containing cell lines, have all of the DDR related proteins that are actively located to sites of viral DNA replication. For example, staining with γH2AX, an indicator of DNA double strand breaks following treatment of cells with external DNA damaging agents, is located at sites of viral DNA replication in the HPV systems [45]. This indicates that there is not widespread host DNA damage in the HPV replicating cells. The fact that the cell progresses through the cell cycle in the presence of the HPV induced DNA damage also suggests that the signaling from ATM and ATR generated by HPV are actually controlled and restricted to the HPV replication foci. If a global signal was sent, then it would be expected that the cell cycle would be arrested. However, there are alternative mechanisms that the virus could use in the presence of the DDR signal generated by viral replication to allow cell cycle progression. The E6 and E7 proteins are oncogenes; they abrogate cell cycle control and are also involved in regulating the DNA damage response by targeting the function of proteins such as p53 and pRb [95]. Therefore, it is possible that the DDR activated via viral replication is overcome by the expression of the viral oncogenes to allow cell cycle progression. However, this cannot be the only mechanism as HPV generated cancer cell lines can still be cell cycle arrested following treatment with exogenous DNA damaging agents. Another possible reason for the controlled response of the cell cycle to HPV replication is that the DDR activated does not seem to be as strong as that generated by traditional DNA damaging agents. For example, the DDR signals generated by E1–E2 replication in C33a cells are not as strong as that generated via the treatment of the cells with etoposide [41]. Another possibility is that the DDR activated by HPV in epithelial cells is different from that generated by traditional DNA damaging agents such as etoposide. It is possible that a combination of all of these mechanisms allows cell cycle progression in the presence of an HPV activated DDR, but the precise reasons and their contributions remain to be elucidated.

**Figure 4:**
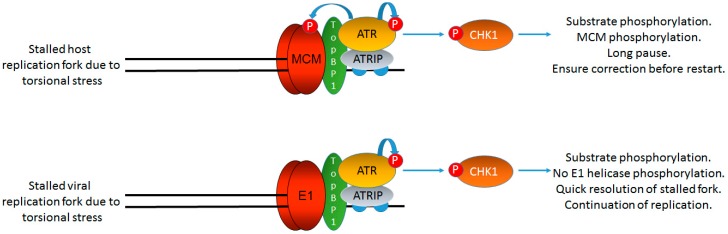
HPV replication is not arrested by DDR signaling. The failure of DDR signaling to arrest E1–E2 mediated DNA replication is essential for the viral life cycle as an active DDR is required for viral replication. However, host cell replication is arrested in the presence of DDR signaling via the phosphorylation of MCM proteins by ATR (and ATM). This uncouples the MCM helicase from DNA polymerases allowing for replication arrest, repair, and restart (top panel). The virus does not have this luxury as it must replicate in the presence of a DDR (bottom panel); therefore, HPV replication has an inherent instability as replication in the presence of external DNA damaging agents is mutagenic as viral replication is not arrested. This mutagenesis would provide substrates for viral genome integration into that of the host, a hallmark of cervical cancer. The DDR signaling generated by E1–E2 replication must be controlled locally to avoid viral arrest of host cell replication via MCM-polymerase uncoupling; such an arrest would be fatal for the viral life cycle. The black lines represent DNA strands. The blue “circular” line represents phosphorylation events.

There is a clearer answer to why E1–E2 replication proceeds in the presence of an active DDR (Figure 4). If the DDR activated is localized to sites of E1–E2 replication, then clearly the virus must be able to replicate in the presence of such signaling. Our work has demonstrated that the activation of the DDR via etoposide has no effect on E1–E2 replication levels even though a cell cycle arrest due to the damage occurs. Similar experiments with the SV40 large T antigen (LT) demonstrated that replication by this protein is arrested following the activation of the DDR via etoposide treatment [96]. The major difference between E1–E2 and LT replication in this study was that LT was a substrate for the ATM/ATR signaling pathways following etoposide treatment, while E1 was not. Therefore, it seems that the HPV viral helicase does not receive signals that would arrest its function in a DNA damaging environment. This would explain why E1–E2 replication can progress in the presence of an active DDR. Significantly, MCM (minichromosome maintenance) proteins are substrates for DDR signaling pathways and phosphorylation uncouples MCM from replicative helicases to arrest host replication in response to the DDR signaling [97,98]. The arrest of host replication will promote high fidelity replication as replication will restart following DNA repair. For the virus, there is a balance and it has to replicate in the presence of a DDR; therefore, it cannot respond with replication arrest to local DDR signaling generated by viral replication. However, the E1–E2 replication carried out in the presence of etoposide is highly mutagenic, and therefore, although the levels of replication are not affected by external DNA damaging treatments, the quality of that replication can be compromised. This is likely due to etoposide induced double stranded breaks during E1–E2 replication in the presence of this damaging agent. Therefore, the virus has an inbuilt genomic instability mechanism: the virus has to replicate in the presence of an active DDR and is therefore not shut down following the exposure of cells to external DNA damaging agents. Replication in the presence of such agents would promote genome breakage. Such breakage would generate substrates for insertion of the viral genome into the host genome via non-homologous end joining. The virus must also replicate DNA that is subject to normal endogenous DNA damage and failure to repair such damage could also contribute to the breakage of the HPV genome and therefore promote integration. Additionally, it has recently been demonstrated that the E6 and E7 proteins can interfere with homologous recombination and this could also contribute to mutagenic E1–E2 DNA replication [99]. It is of note that in C33a cells E2 locates to fragile sites [100]. Viral replication at such sites in HPV infected cells would mean that breakage of the viral genome near such sites would facilitate viral genome integration into that of the host. Such integration is an indicator of a worse clinical outcome in cervical cancer. Therefore, the action of external DNA damaging agents could promote viral breakage and integration into the host genome, a hallmark of cervical cancer. Correspondingly, the human body is constantly exposed to chemical compounds that can act as DNA damaging agents including estrogens and xenoestrogens. Epidemiological studies, as well as transgenic mouse models, have suggested a significant role of estrogen in the development of cervical cancer, and estrogen receptor α (ERα) has been shown to be required for the development of HPV-oncogene-induced cervical neoplasia and cancer in mice [101,102,103,104]. In the mouse models (where no E1–E2 mediated viral replication is occurring), this is due to the interaction of the ER receptor with the stromal tissue, but how estrogen might contribute to cervical cancer in humans is not fully understood. While HR-HPV is necessary but not sufficient for malignant transformation, it is possible that estrogen may contribute to cancer development via the initiation of the DDR in HPV infected cells, promoting viral breakage and integration [105,106,107]. Estrogen also diminishes ATR and *TopBP1* association following DNA damage, as well as Claspin/Chk1 protein association via PI3K/AKT signaling; this disruption could promote the dissolution of E1–E2 replication foci promoting mutagenic viral replication, as we have already demonstrated following *TopBP1* depletion [41,108]. Such replication would provide substrates for integration into the host genome. Estrogen also prolongs γH2AX and Rad51 nuclear foci, delays DNA repair, and can increase chromosomal damage in cells exposed to DNA damaging agents such as ionizing radiation [108]. Therefore, altering DDR protein interactions, as well as prolonging damage signals, could contribute to HPV integration.

## 8. Conclusions

Several DNA viruses actively suppress the host DDR in order to promote their viral life cycles. So why does HPV promote an active DDR in infected cells? We propose that the answer lies in the nature of replication during the viral life cycle. During the establishment and amplification phases of the viral life cycle, the circular 8 kb viral genome has to replicate more than once per cell cycle. This replication would place stress on the viral genomes that would generate single stranded DNA and double stranded breaks that would activate the DDR pathways. Without this activation, the virus could not successfully replicate and decatenate its genome. It has been proposed that the recruitment of a host of homologous recombination (HR) proteins to the viral genome allows a switch in viral replication at the amplification stage of the viral life cycle using an HR mechanism [109]. It has also been shown that there is a switch to a rolling circle mechanism of replication that promotes the amplification of the viral genome in differentiated HPV containing cells [110]. Neither of these mechanisms are inconsistent with the idea that the viral replication process per se is able to activate the DDR. The “onion skin” like replication structures that the virus generates would be recognized by the cell as DNA damage. This would recruit factors such as *TopBP1* and activate the ATR and ATM pathways locally, further stimulating the DDR. Therefore, the reason that the DDR is initially turned on by HPV probably involves the resolution of DNA structures during the establishment phase of the viral life cycle. Using HR to resolve these structures would generate wild type viral genomes. It is possible that the virus could replicate an entire genome before beginning the replication again, therefore avoiding onion skin like structures. We do not favor this model as it is hard to imagine such a complex mode of replication, constantly switching the E1 and E2 function on and off, and this would also provide multiple fail points for the viral life cycle. Such replication would also not induce a DDR. DDR pathways remain turned on even during the maintenance phase of the viral life cycle, although it is noticeable that this pathway is not required for maintenance phase replication. The reason that the DDR may be retained in the HPV infected cells could relate to the amplification stage of the viral life cycle. Whatever the mechanism that promotes amplification of the viral genome in the infected cells, it is likely that as the viral genome copy number increases from 20–50 to around 1000 copies per cell, aberrant viral DNA structures are again generated. Therefore, a primed DDR signaling pathway could promote this amplification stage of the viral life cycle and HR would help produce healthy viral genomes for encapsulation by L1 and L2. The amplification of the viral genomes in discrete nuclear foci could also facilitate the formation of the viral particles. If L1 and L2 had to “search” throughout the cell for the viral genomes during the late stages of the viral life cycle in order to encapsulate them, this would be a very inefficient process. It is known that there is an interaction between E2 and L2; therefore, the presence of E2 in the viral replication factories could facilitate L1 and L2 recruitment to the viral genomes to promote encapsulation and viral particle egress [111].

Much work remains to be carried out in this field of HPV research. We do not fully understand what controls the three phases of replication during the viral life cycle and what the precise contribution of DDR proteins are in each step. Future work will focus on elucidating these processes, as an enhanced understanding of them could identify targets for disrupting the viral life cycle.
